# The synthesis, thermal behaviour, spectral and structural characterization, and in silico prediction of pharmacokinetic parameters of tetraalkylammonium salts of non-steroidal anti-inflammatory drug nimesulide

**DOI:** 10.1038/s41598-023-44557-x

**Published:** 2023-10-12

**Authors:** Małgorzata Rybczyńska, Artur Sikorski

**Affiliations:** Faculty of Chemistry, University of Gdańsk, W. Stwosza 63, 80-308 Gdańsk, Poland

**Keywords:** Crystal engineering, Chemistry

## Abstract

The synthesis, spectral properties, thermal analysis, structural characterization and in silico prediction of pharmacokinetic parameters of tetramethylammonium (compound **1**) and tetraethylammonium (compound **2**) salt of nimesulide were described in this article. Both compounds crystallize in the monoclinic *P*2_1_/n space group, with one tetraalkylammonium cation and one nimesulide anion in the asymmetric unit and their crystal structures are stabilized by C–H···O hydrogen bonds between ions. Additionally, structures of title compounds are stabilized by π–π interactions (compound **1**), or C–H···π interactions (compound **2**) between nimesulide anions. The TG and DSC measurements show that compound **1** melts at a temperature higher than nimesulide, whereas the compound **2** melts at a temperature lower than nimesulide. The MALDI-TOF, ^1^H NMR, ^13^C NMR and ATR-FTIR analyses confirm the SCXRD study, that in compounds **1** and **2** nimesulide exists in an ionized form. Studies performed by SWISS ADME and ProTOX II tools, predict to be oral bioavailability of both salts obtained, and one of them (compound **1**) is predicted to be well-absorbed by digestive system, while both compounds obtained are classified into toxicity class 4.

## Introduction

The development of crystal engineering makes it possible to obtain new forms of drugs^1–8^. This is possible, among other things, due to the understanding of intermolecular interactions between the molecules or ions, which enables the design of multi-component crystals involving Active Pharmaceutical Ingredients (API). Obtaining multi-component crystals using APIs makes it possible to improve the physicochemical properties of drugs such as thermal stability, lipophilicity and solubility, which allows for, among others, various possibilities of drug administration, reducing the dose of the drug substance and extending the time interval between drug dosing^9–13^. Determining the structures of such crystals using XRD methods makes it possible to determine the correlation between their structure and properties such as melting point, absorption, or reactivity^14–18^. Crystallization of multi-component crystals can also improve the narrow therapeutic index of drugs^9–18^. What is more, pharmacodynamic interactions between two or more bioactive substances can change properties and pharmaceutical action of different drugs which is called synergism^19–22^. Such a combination causes that properties one or both drugs may be greatly strengthened, or substances may have an antagonistic action^19–22^.

**Figure 1:**
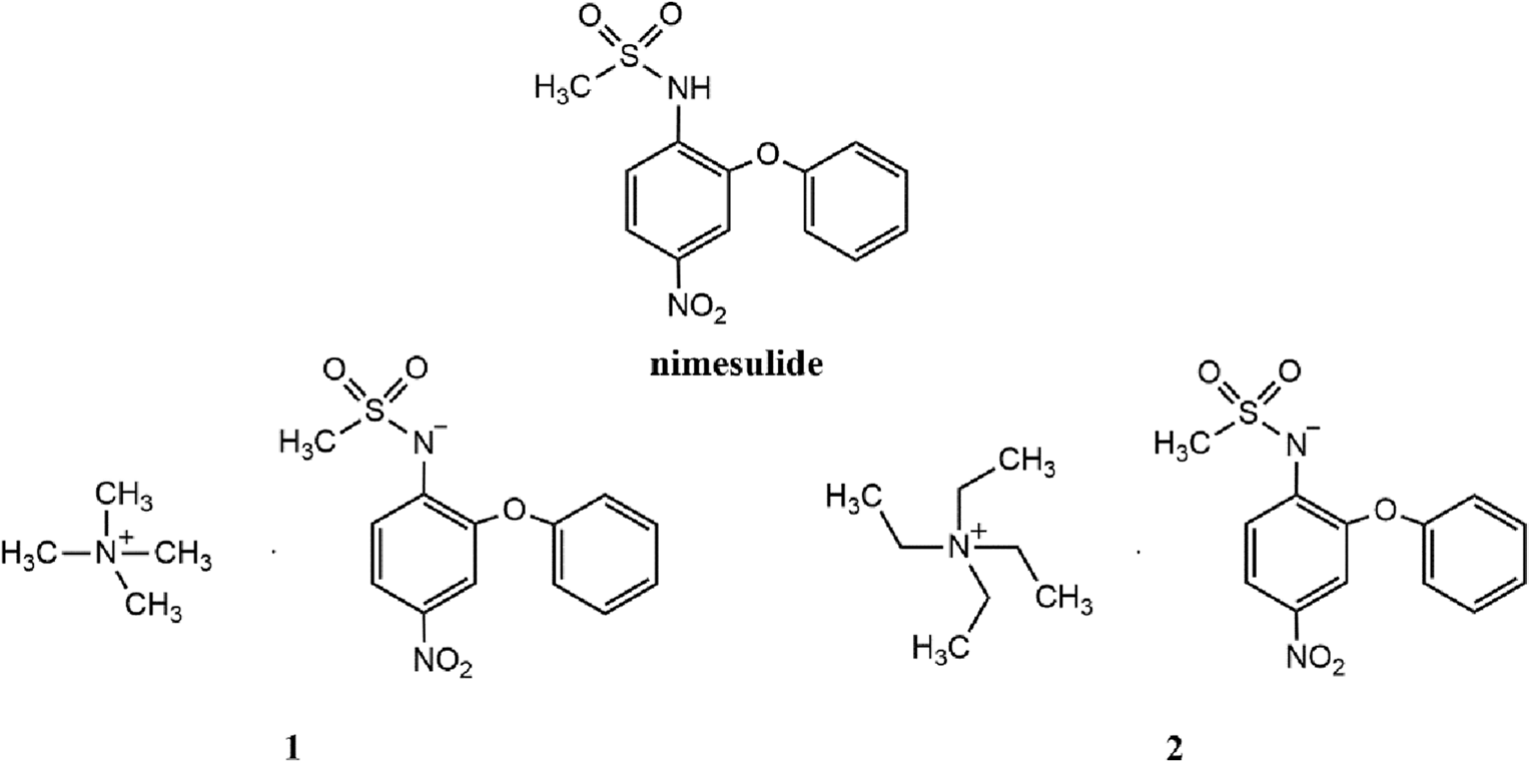
The molecular structures of nimesulide and tetramethylammonium (compound **1**) and tetraethylammonium (compound **2**) salts of nimesulide.

An interesting example of multicomponent crystals of pharmaceutical interest are crystals containing two or more bioactive substances, such as nimesulide and quaternary ammonium cations. Nimesulide (4-nitro-2-phenoxymethanesulfonanilide) is an Active Pharmaceutical Ingredient belongs to the non-steroidal anti-inflammatory drugs (NSAIDs) (Fig. 1). It has antipyretic, analgesic and anti-inflammatory properties and it is use in acute pain treatment and primary dysmenorrhea. Like most NSAIDs, the mechanism of action of substances is based on the inhibition of cyclooxygenase—an enzyme that is involved in the synthesis of prostaglandins from lipids in cell membranes^23–26^. In turn, tetramethylammonium cation is a cholinomimetic that imitate effects as acetylcholine and causes stimulation and then block of neurotransport nicotinic and muscarinic acetylcholine receptors^27–30^, whereas tetraethylammonium anion is ganglionic blocker and is inhibitor at nicotinic acetylcholine receptors^31,32^.

The structures of crystals involving nimesulide are poorly known—in the Cambridge Structural Database (CSD ver. 5.41, update 01.2023) there are only 11 such structures^33^. Two of them are the structures of two nimesulide polymorphs determined at room and low temperatures (100 K)^26,34,35^, five structures are complexes of silver with nimesulide and various ligands^35^, whereas four structures are cocrystals of nimesulide with pyridine analogues^36^. In the CCDC there are also information about inclusion complex of nimesulide with cyclodextrin, however the crystal structure of this compound was not determined^37,38^.

Considering the above, in this article we describe the synthesis, spectral and structural characterization of tetramethylammonium (compound **1**) and tetraethylammonium (compound **2**) salts of nimesulide (Fig. 1). The spectral characterization of compounds obtained were studied by MALDI-TOF, ^1^H NMR, ^13^C NMR and ATR-FTIR methods, whereas the crystal structures were determined using the Single-Crystal X-Ray Diffraction method. Physicochemical features of compounds obtained were predicted in silico using SWISS ADME and Protox II tools.

## Experimental

### Materials

Tetramethylammonium hydroxide pentahydrate and tetraethylammonium hydroxide solution were purchased from Sigma-Aldrich, whereas nimesulide was received from Ambeed and they were used without further purification. The purity of nimesulide and compounds **1** and **2** and were controlled by TG/DSC methods.

### Synthesis of compound 1

Nimesulide (0.05 g, 0.162 mmol) and tetramethylammonium hydroxide pentahydrate (0.026 g, 0.162 mmol) were dissolved in the mixture of solvents: 5 cm^3^ of ethanol and 5 cm^3^ of a methanol. After this, the mixture was stirred. The solution was allowed to evaporate in place without sunlight for a few days to give yellow crystals (m.p. = 179 °C).

**Figure 2:**
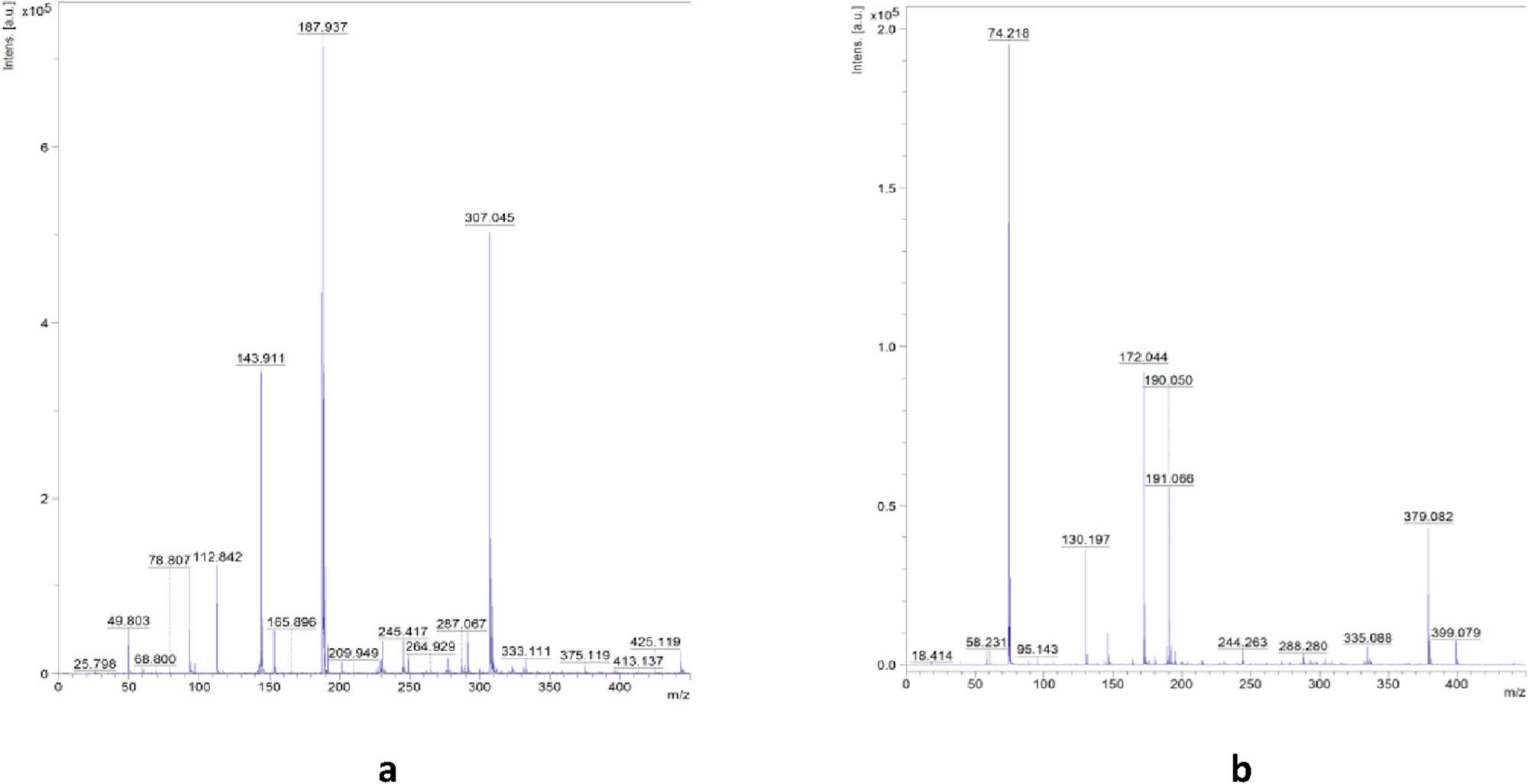
MALDI-TOF mass spectra for compound **1** in negative-ion (**a**) and positive-ion (**b**) mode.

MALDI-TOF MS (*m/z*): calc. for [C_13_H_11_N_2_O_5_S]^-^: 307.303; found: 307.045 (in negative-ion mode, Fig. 2a); calc. for [C_4_H_12_N]^+^: 74.144; found: 74.218 (in positive-ion mode, Fig. 2b).

^1^H NMR (500 MHz, DMSO-d_6_) δ (ppm): 7.83 (dd, *J* = 9.3, 2.8 Hz, 1H), 7.50 (d, *J* = 2.8 Hz, 1H), 7.35 (d, *J* = 9.3 Hz, 1H), 7.30 (dd, *J* = 8.6, 7.3 Hz, 2H), 7.03 (tt, *J* = 7.3, 1.2 Hz, 1H), 6.87 (dd, *J* = 8.6, 1.2 Hz, 2H), 3.09 (s, 12H), 2.62 (s, 3H) (Fig. 4a).

^13^C NMR (126 MHz, DMSO-d_6_) δ (ppm): 158.23, 152.15, 145.56, 134.05, 129.93, 122.34, 122.29, 117.40, 117.33, 116.25, 54.86 (1:1:1 triplet by coupling with ^14^N), 40.80 (Fig. 4b).

ATR-FTIR (/cm): 3098 and 3059 (νC–H_aromatic_), 2993 (νC–H_aliphatic_) 1582–1467 (νC=C and ν_as_NO_2_), 1320 and 1165 (shoulder low-intensity bands, ν_as_ and ν_sym_SO_2_), 1280–1070 (νC–N and νC–O) (Fig. 5).

### Synthesis of compound 2

Nimesulide (0.05 g, 0.162 mmol) was dissolved in 0.12 ml tetraethylammonium hydroxide solution (20 wt. % in H_2_O, d = 1.01 g/cm^3^ in 20 °C, 0.162 mmol). After this, the mixture was stirred. The solution was allowed to evaporate in place without sunlight for a few days to give yellow crystals (m.p. = 113 °C).

**Figure 3:**
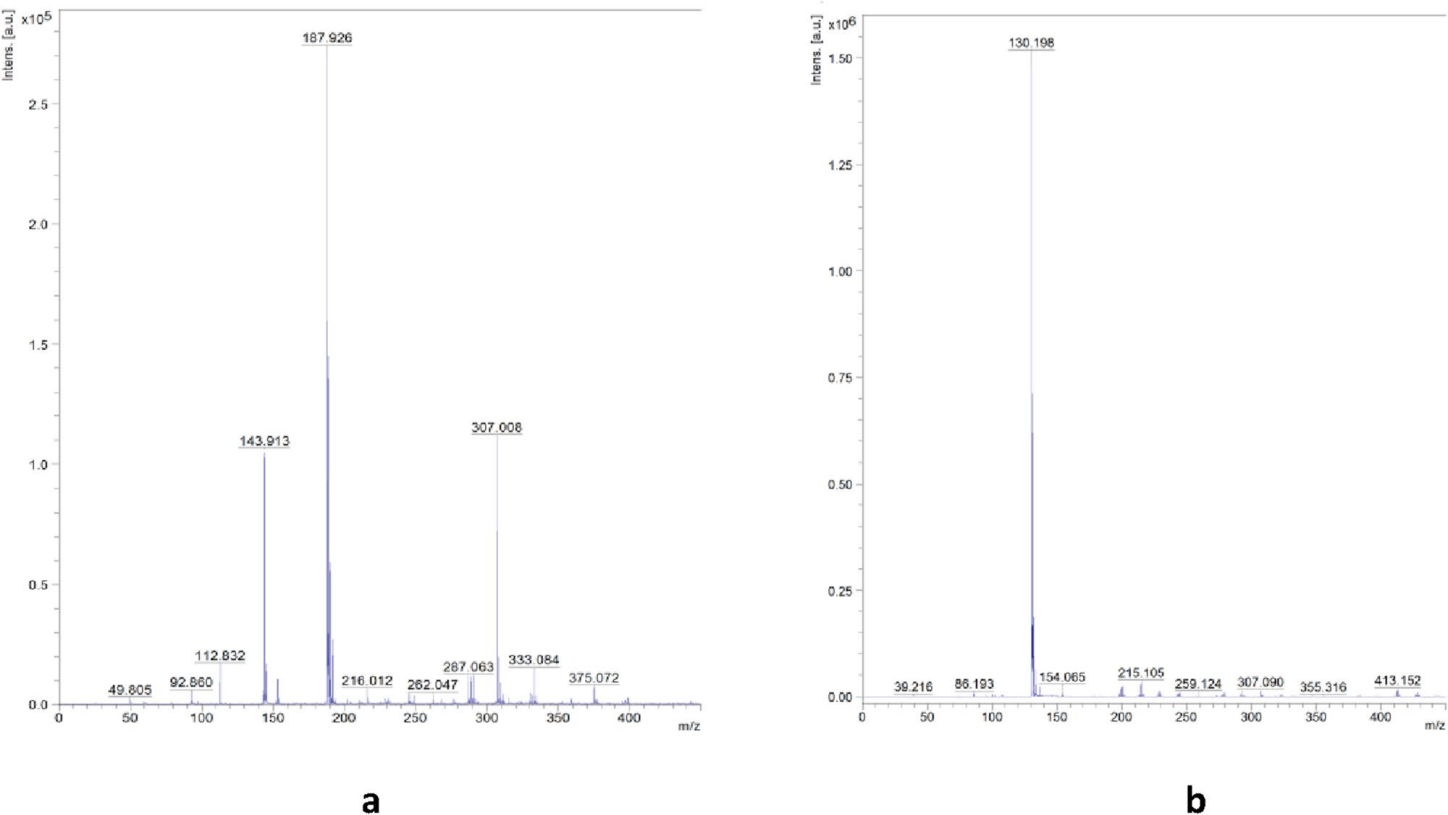
MALDI-TOF mass spectra for compound **2** in negative-ion (**a**) and positive-ion (b) mode.

MALDI-TOF MS (*m/z*): calc. for [C_13_H_11_N_2_O_5_S]^−^: 307.303; found: 307.008 (in negative-ion mode, Fig. 3a); calc. for [C_8_H_20_N]^+^: 130.250; found: 130.198 (in positive-ion mode, Fig. 3b).

**Figure 4:**
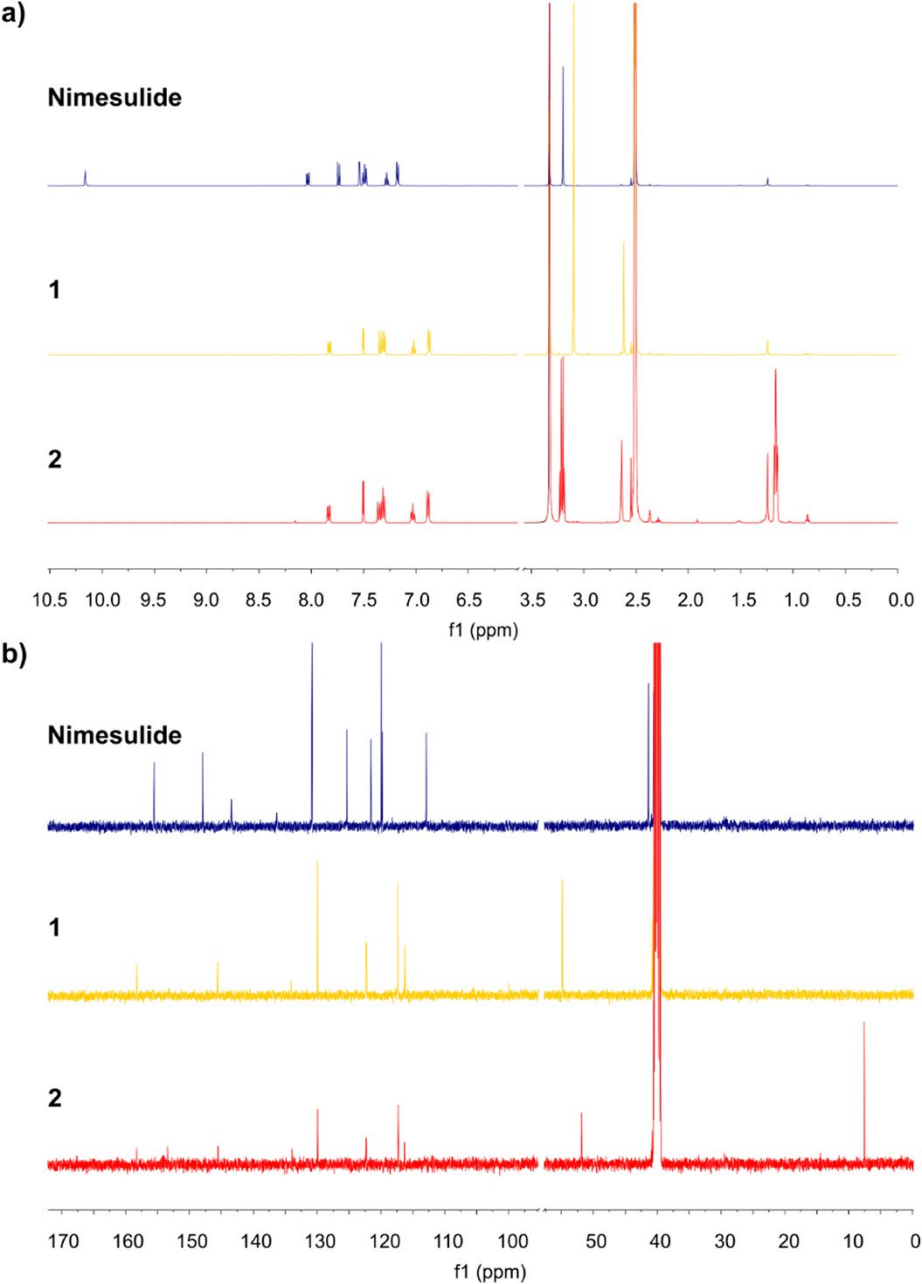
^1^H NMR (**a**) and ^13^C NMR spectra (**b**) of nimesulide and compounds **1** and **2** in DMSO-d_6_ at 298 K.

^1^H NMR (500 MHz, DMSO-d_6_) δ (ppm): 7.84 (dd, *J* = 9.3, 2.8 Hz, 1H), 7.50 (d, *J* = 2.8 Hz, 1H), 7.37 (d, *J* = 9.3 Hz, 1H), 7.31 (dd, *J* = 8.3, 7.2 Hz, 2H), 7.03 (t, *J* = 7.2 Hz, 1H), 6.88 (d, *J* = 8.3 Hz, 2H), 3.21 (q, *J* = 7.3 Hz, 8H), 2.64 (s, 4H), 1.16 (m, ^3^J_HH_ = 7.3, J_14N,CH3_ = 1.8 Hz, 12H) (Fig. 4a).

^13^C NMR (126 MHz, DMSO-d_6_) δ (ppm): 158.27, 153.43, 145.52, 133.92, 129.92, 122.32, 122.29, 117.34, 117.29, 116.30, 51.84 (1:1:1 triplet by coupling with ^14^N), 40.80, 7.54 (Fig. 4b).

**Figure 5:**
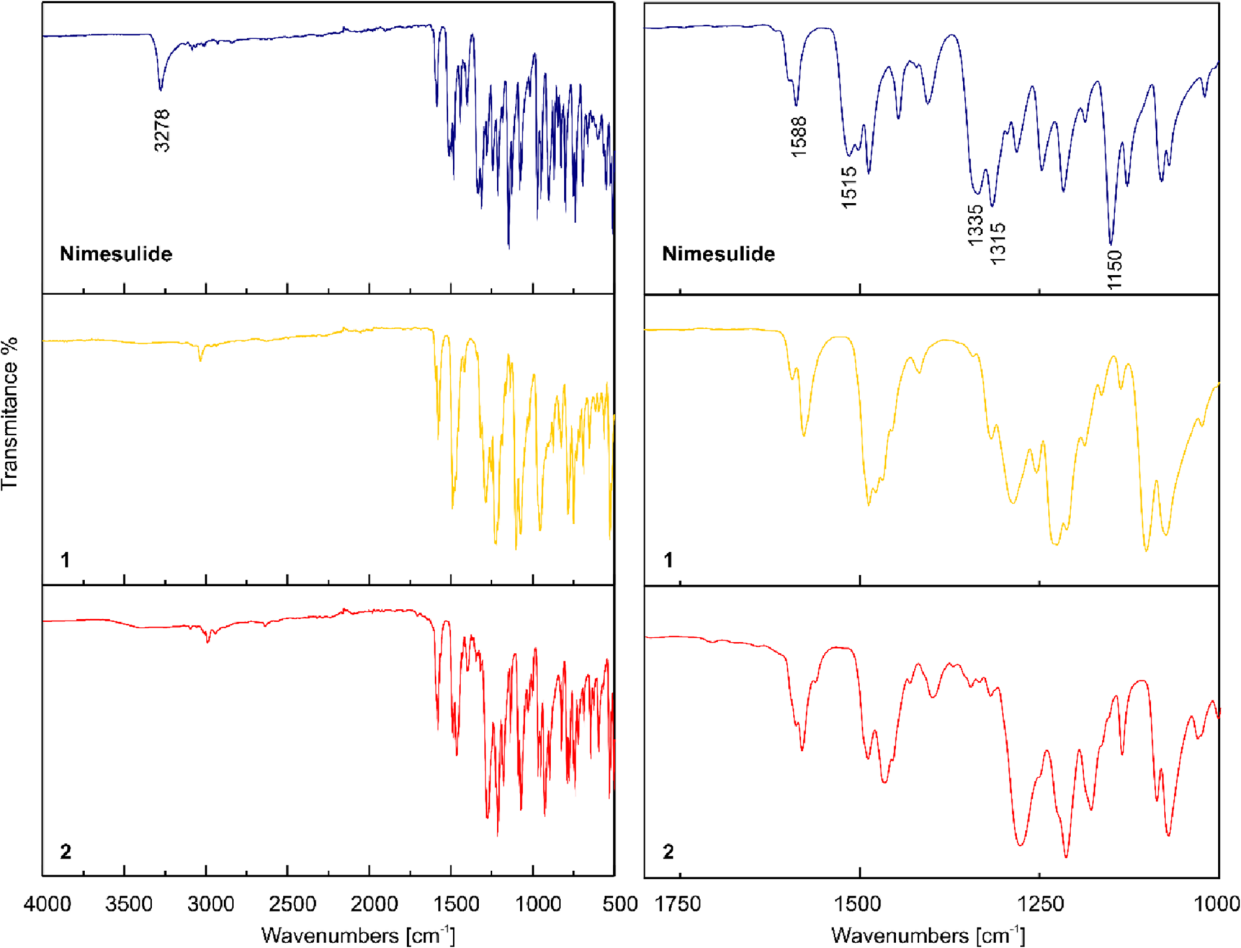
ATR-FTIR spectra of studied compounds in the range of 4000–500/cm (left panel) with spectra enlargement in the range of 1800–1000/cm (right panel).

ATR-FTIR (/cm): 3032 (νC–H_aromatic_), 1577–1468 (νC=C and ν_as_NO_2_), 1318 and 1163 (shoulder low-intensity bands, ν_as_ and ν_sym_SO_2_), 1280–1073 (νC–N and νC–O) (Fig. 5).

## Methods

### MALDI-TOF mass spectra

Positive-ion and negative-ion mode MALDI-TOF mass spectra for compounds **1** and **2** were obtained using a Bruker Biflex III spectrometer with α-cyano-4-hydroxycinnamic acid matrix in positive- and negative-ion mode.

### Nuclear magnetic resonance (NMR)

The ^1^H NMR and ^13^C NMR spectra were recorded on a Bruker III Avance 500 MHz spectrometer (^1^H frequency 500.13 Hz) operated at magnetic fields of 11.7 T, using standard experimental conditions in DMSO-d_6_ solution.

### Attenuated Total Reflectance-Fourier Transform Infrared Spectroscopy (ATR–FTIR)

The ATR-FTIR spectra were acquired using a Perkin Elmer Spectrum 2™ instrument (Perkin Elmer, Waltham, USA) equipped with attenuated total reflectance (ATR) accessory. The spectra were recorded at room temperature in the spectral range from 4000 to 500/cm at a resolution of 4/cm averaging 16 scans for each measurement.

### Thermogravimetry (TG) and differential scanning calorimetry (DSC)

Thermogravimetric measurements (TG) were performed with a Netzsch 209 thermobalance: samples weighing ~ 4.0 mg were placed in a platinum crucible and heated at 10.0 K/min in a dynamic Ar atmosphere. Differential scanning calorimetry (DSC) measurements were performed with a Perkin Elmer TGA 8000 instrument: samples weighing ~ 4.0 mg were placed in an aluminium crucible and heated at 10.0 K/min in a dynamic N atmosphere.

### Single-Crystal X-Ray Diffraction (SCXRD) measurements and structure refinement

SCXRD data were collected on an Oxford Diffraction Gemini R ULTRA Ruby CCD diffractometer MoKα (*λ*_Mo_ = 0.71073 Å, T = 293(2) K) (Table 1). The lattice parameters were obtained using CrysAlis CCD, while data were reduced using CrysAlis RED software (multi-scan absorption corrections were applied)^39^. Crystal structures were solved by direct methods using SHELXS-97 and refined by full-matrix least-squares on *F*^2^ with anisotropic displacement parameters for non-H atoms using SHELXL-2017/1^40^. All H–atoms were placed geometrically and refined using a riding model with C–H = 0.93 ÷ 0.97 Å and U_iso_(H) = 1.2U_eq_(C) (C–H = 0.96 Å and U_iso_(H) = 1.5U_eq_(C) for the methyl groups). The phenoxy group in compound **1** has disordered orientations with refined site-occupancy factors of the disordered parts of 0.533(6) and 0.467(6) (the disordered benzene rings were refined as rigid ideal hexagons with C–C = 1.38 Å and constrained with isotropic displacement parameters). In all figures, the disordered phenoxy group is omitted for clarity. All interactions were found using the PLATON program^41^. The molecular graphics were prepared using ORTEPII^42^, PLUTO-78^43^, and Mercury^44^ software.

**Table 1:** Crystal data and structure refinement for title compounds.

Compound	1	2
Chemical formula	C_17_H_23_N_3_O_5_S	C_21_H_31_N_3_O_5_S
Formula weight/g/mol	381.44	437.55
Crystal system	monoclinic	monoclinic
Space group	*P*2_1_/n	*P*2_1_/n
*a*/Å	13.7425(18)	12.0108(7)
*b*/Å	8.3439(9)	11.5166(7)
*c*/Å	17.529(2)	16.7784(9)
*α*/°	90	90
*β*/°	108.752(14)	97.564(5)
*γ*/°	90	90
*V*/Å^3^	1903.3(4)	2300.6(2)
*Z*	4	4
*T*/K	293(2)	293(2)
*λ*_Mo_/Å	0.71073	0.71073
*ρ*_*cal*c_/g/cm^3^	1.331	1.263
*F(000)*	808	936
Crystal size/mm^3^	0.41 × 0.20 × 0.09	0.44 × 0.21 × 0.09
µ/mm	0.202	0.176
*θ* range/°	3.30–25.00	3.63–25.00
Completeness *θ*/%	99.8	99.7
Reflections collected	11,775	15,981
Reflections unique	3328 [R_int_ = 0.0481]	4043 [R_int_ = 0.0285]
Data/restraints/parameters	3328/66/269	4043/0/276
Goodness of fit on *F*^*2*^	1.039	1.087
Final R_1_ value (*I* > 2σ(*I*))	0.0785	0.0597
Final *w*R_2_ value (*I* > 2σ(*I*))	0.1763	0.1605
Final R_1_ value (all data)	0.1275	0.0767
Final *w*R_2_ value (all data)	0.2084	0.1729
Largest diff. peak/hole/e/Å^3^	0.226/− 0.227	0.499/− 0.227
CCDC number	2,281,374	2,281,375

### ADME and Protox II analyses

The web-service SWISS-ADME tool by the Swiss Institute of Bioinformatics (http://www.swissadme.ch/) was used to calculate physicochemical descriptors, important for drug discovery^45^. Compounds were analyzed to predict ADME (absorption, distribution, metabolism, and excretion) parameters, pharmacokinetic properties, or drug-like nature in comparison to pure nimesulide. The web-service ProTOX II was used for the prediction of the toxicity of the title compounds^46^.

## Results and discussion

### Spectral characterization

Negative-ion and positive-ion mode MALDI-TOF mass spectra for compounds **1** and **2** are shown in Figs. 2 and 3. For compounds **1** and **2**, the intensity for nimesulide anion were found at *m/z* 307.045, and 307.008, respectively (calculated for [C_13_H_11_N_2_O_5_S]^−^: 307.303), whereas the intensity for tetramethylammonium and tetraethylammonium cations appeared at *m/z* 74.218 and *m/z* 130.198, respectively (calculated for [C_4_H_12_N]^+^: 74.144 and calculated for [C_8_H_20_N]^+^: 130.250). The differences between intensities found and calculated are insignificant.

The ^1^H and ^13^C NMR spectra of pure nimesulide, and compounds **1** and **2** are shown in Fig. 4. The ^1^H and ^13^C NMR data are presented in the “Experimental” section.

Chemical shifts, multiplicities and the observed proton-proton coupling constants are in accordance with the structure of the studied compounds. The number of carbon signals corresponds to the expected number of carbon atoms of the studied compounds. A singlet at 10.2 ppm in the ^1^H NMR spectra of pure nimesulide indicates N–H proton of sulfonamide group^47^. This resonance disappeared in the ^1^H NMR spectra of compounds **1** and **2** due to the amide proton detachment (Fig. 4a). Resonances between 6.5 and 8.5 ppm correspond to aromatic protons. NMR resonances with chemical shifts in the range of 1–3.5 ppm correspond to aliphatic protons of the methylene and methyl groups. The signals in the range of 110–160 ppm in the ^13^C NMR spectra indicate aromatic carbons, whereas those in the range of 0–55 ppm correspond to saturated functional groups (Fig. 4b).

ATR-FTIR spectra of pure nimesulide and compounds **1** and **2** are presented in Fig. 5. The ATR-IR spectrum of the pure nimesulide shows characteristic bands at 3278/cm (N–H stretch), 1587/cm (aromatic rings), 1335/cm and 1150/cm (asymmetric and symmetric stretching vibrations of SO_2_ group), 1515/cm and 1315/cm (asymmetric and symmetric stretching vibrations of aryl NO_2_ group). These values are in agreement with literature results ^26,34,48–50^.

Formation of the salts results in a disappearance of N–H band, which is related to proton detachment from N-amine atom. The changes are also observed within the stretching vibrations of SO_2_ and NO_2_ groups. The asymmetric and symmetric stretching vibrations of SO_2_ group are observed at slightly lower wavenumbers as shoulder low-intensity bands for both salts, whereas those of nitro group remain strong but are shifted to lower wavenumbers by 25–35/cm.

### TG/DSC studies

**Figure 6:**
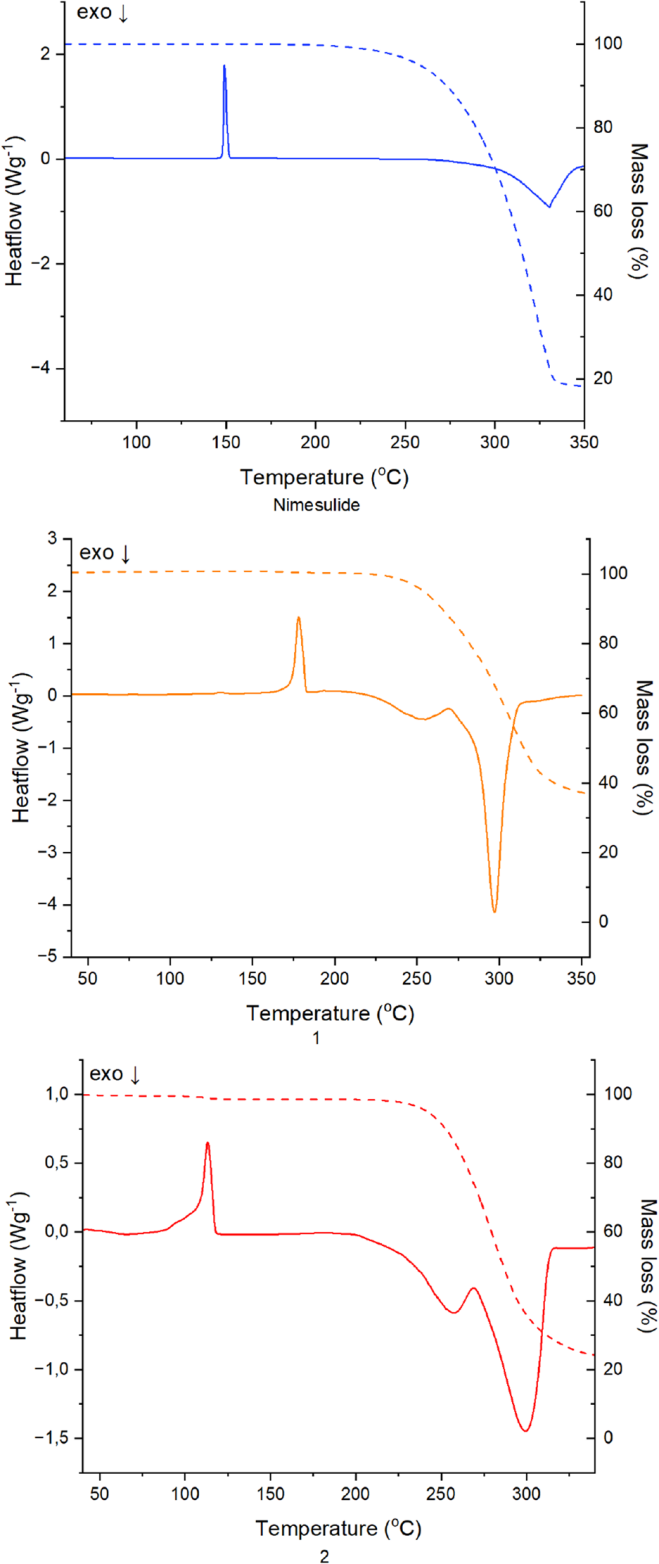
DSC-TG curves of nimesulide and compounds **1** and **2**.

The thermal behaviour of pure nimesulide and compounds **1** and **2** are shown in Fig. 6. The TG-DSC diagrams for the nimesulide show a sharp endothermic peak at 149 °C, which represent the melting point, whereas the first decomposition step occurs between 220 and 390 °C with a mass loss of 71.9% associated with an exothermic peak at 330 °C attributed to the decomposition of the compound. These temperatures are in agreement with literature results ^26,34,48–50^. The TG-DSC curves for compounds **1** and **2** exhibit sharp endothermic peaks at 179 and 113 °C, which represent the melting points of both compounds, respectively. For the compound **1**, the first decomposition step occurs between 219 °C and 377 °C with a mass loss of 64.7% associated with exothermic peaks at 269 and 297 °C. For the compound **2**, the first decomposition step is observed in range of 205–360 °C with a mass loss of 75.4% associated with exothermic peaks at 258 and 297 °C.

### Single-Crystal X-Ray Diffraction (SCXRD) studies

**Figure 7:**
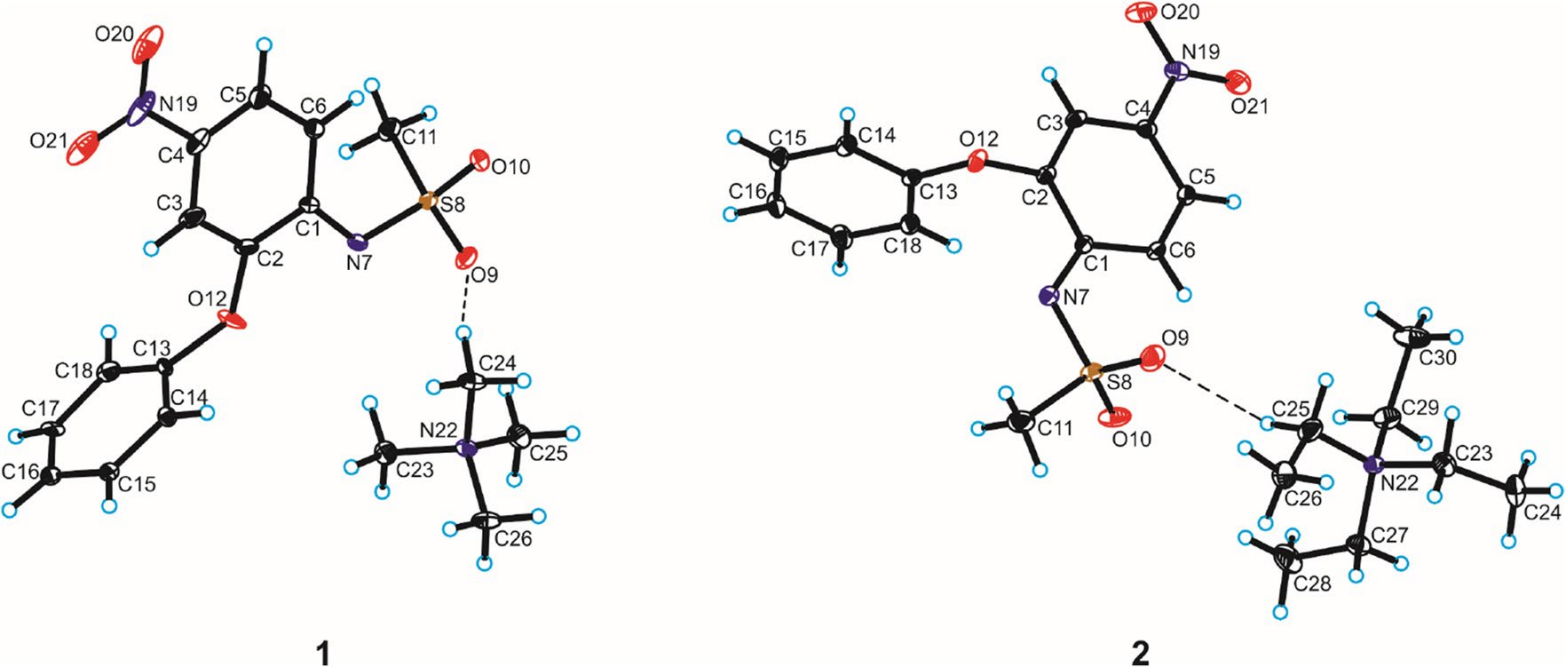
Crystal structures of compounds **1** and **2** with the atom-labelling scheme (hydrogen bonds are represented by dashed lines).

Compounds **1** and **2** crystallize in the monoclinic *P*2_1_/n space group with one tetraalkylammonium cation and one nimesulide anion in the asymmetric unit (Fig. 7, Table 1).

In both crystal structures, the proton detachment from N7-atom of nimesulide is observed which is confirmed by *d*(C–N) and *d*(N–S) bond length and ∠(C–N–S) angle within the sulfonamide group. In the crystals of title compounds, the values of *d*(C–N) bond length are 1.37 and 1.35 Å, whereas the *d*(N–S) bond length are 1.56 and 1.58 Å for compounds **1** and **2**, respectively. For comparison, in the crystal structures of nimesulide (form I and II) the *d*(C-N) bond length is 1.40 and 1.41 Å, whereas the *d*(N–S) bond length are 1.64 and 1.65 Å, respectively for form I and II^26,34–36^. In turn, in the crystals of complexes of silver with nimesulide the *d*(C–N) bond length is in the range 1.38 ÷ 1.40 Å, while the *d*(N–S) bond length is in the range 1.38 ÷ 1.62 Å^35^. For the structures of cocrystals of nimesulide with pyridine analogues, the values of these bond length are 1.42 and 1.64 Å, respectively^37^. In the crystals of title compounds, the values of ∠(C–N–S) angle are 122.2° and 119.2°, for compounds **1** and **2**, respectively, which indicates the *sp*^2^ hybridization of the nitrogen atom. For comparison, in the crystal structures of nimesulide the ∠(C–N–S) angle is 124.6 and 124.1°, for form I and II, respectively^26,34–36^. In turn, in the crystals of complexes of silver with nimesulide the ∠(C–N–S) angle is in the range 118.7 ÷ 122.3°^35^, whereas for the structures of cocrystals of nimesulide with pyridine analogues, this angle is in the range 119.9 ÷ 124.0°^37^.

**Figure 8:**
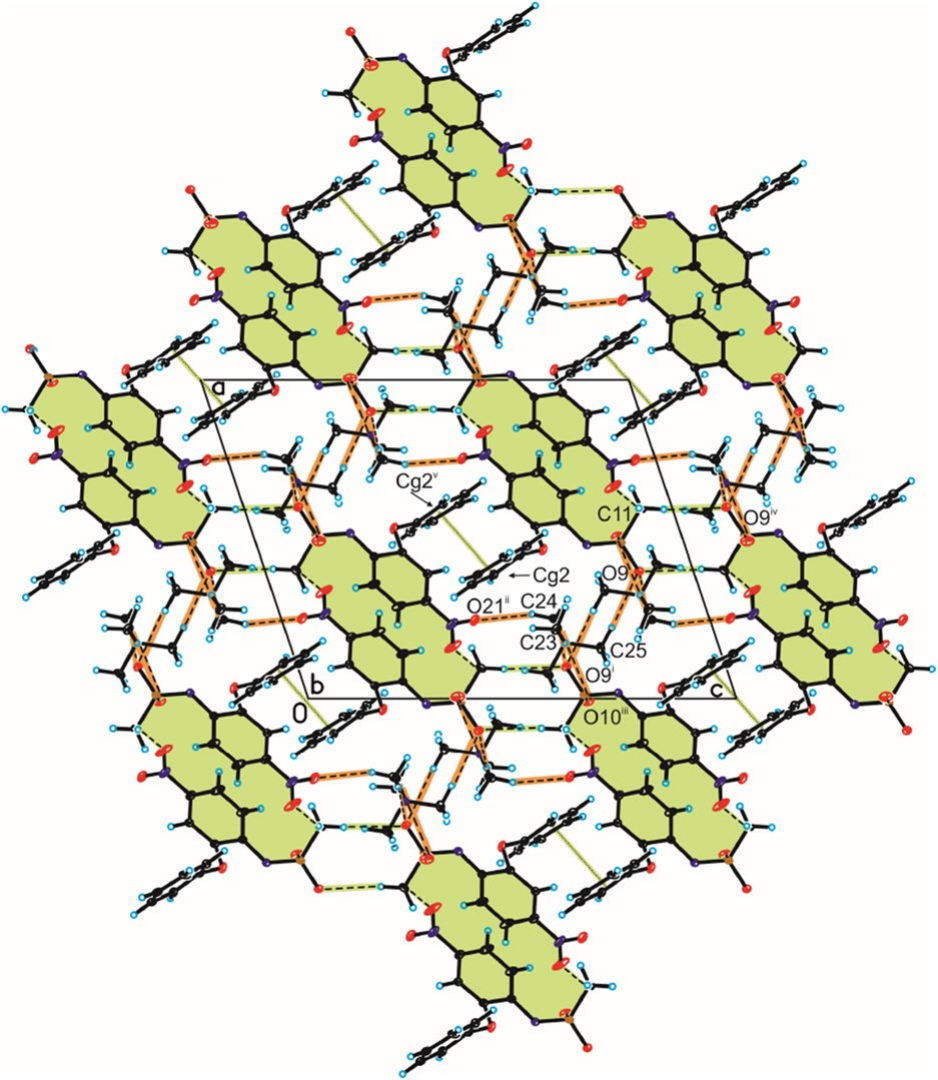
Crystal packing of compound **1** viewed along *b*-axis (interactions between nimesulide anions are highlighted by green, whereas hydrogen bonds between nimesulide anion and tetramethylammonium cation are highlighted by orange).

In the crystal of compound **1**, the nimesulide anions are linked by π–π interactions between aromatic ring of phenoxy groups [*d*(Cg···Cg) = 3.374(6) Å and offset = 1.496 Å] (Fig. 8, Table S3) to form homodimer. The neighbouring homodimers are linked through C_(methyl)_–H···O_(nitro)_ hydrogen bond, building blocks along [1 0 1] direction (Fig. 8, Table S2). The adjacent blocks of nimesulide anion are connected by C_(methyl)_–H···O_(sulfo)_ hydrogen bond to create 3D framework with channels (Fig. 8, Table S2). In these channels the tetramethylammonium cations are located and interact with the nimesulide anions by weak C_(methyl)_–H···O_(nitro)/(sulfo)_ hydrogen bonds.

**Figure 9:**
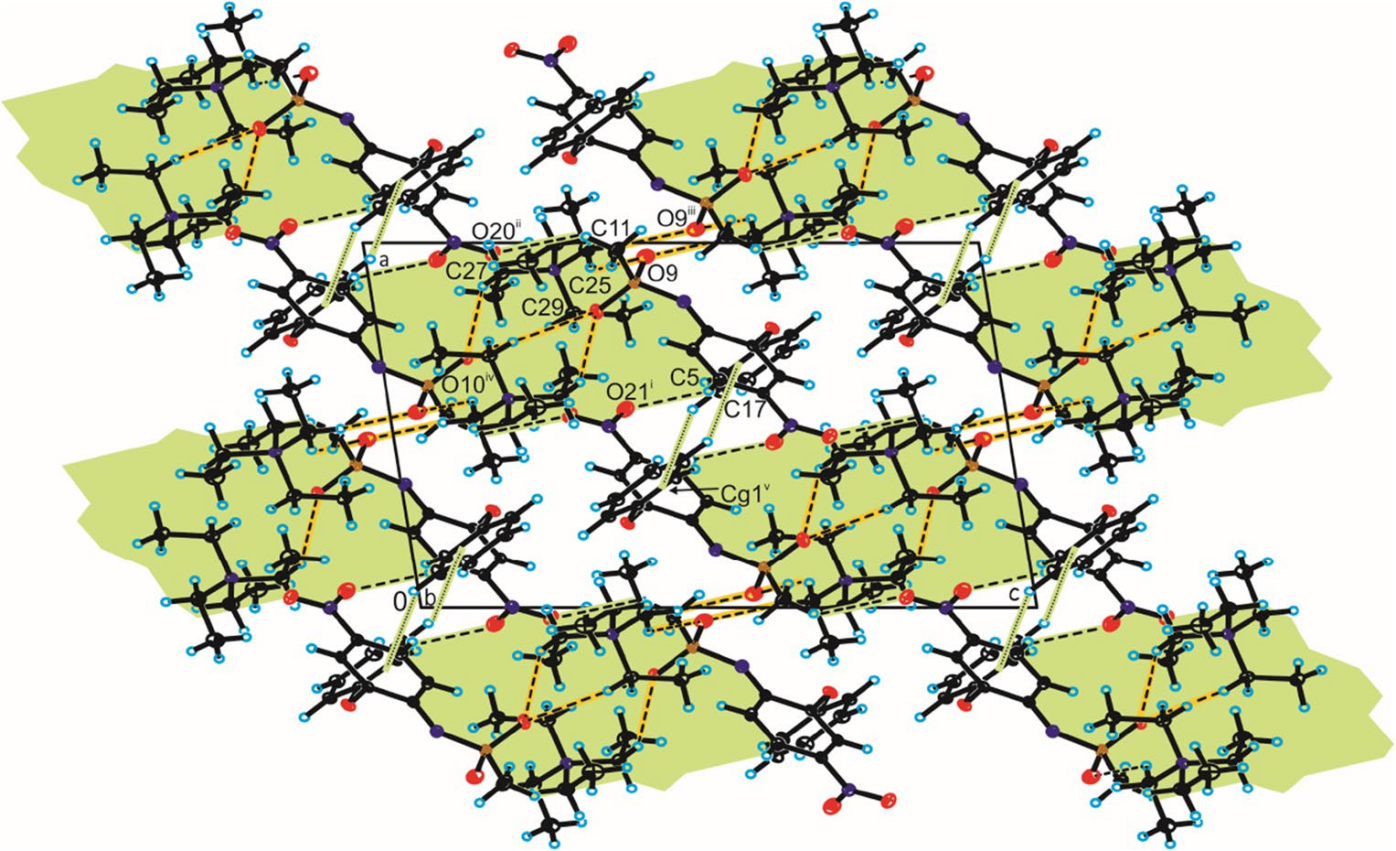
Crystal packing of compound **2** viewed along *b*-axis (interactions between nimesulide anions are highlighted by green, whereas hydrogen bonds between nimesulide anion and tetraethylammonium cation are highlighted by orange).

In the crystal of compound **2**, the homodimer of anions is formed by C–H···π interactions (*d*(H···Cg) = 2.97 Å) between both aromatic rings of nimesulide (Fig. 9, Table S4). The neighbouring dimers are linked by C_(methyl)_–H···O_(nitro)_ hydrogen bond, building columns along *b*-axis (Fig. 9, Table S2) in voids of which tetraethylammonium cations are located and linked with nimesulide anions via C_(methyl)_–H···O_(nitro)_ hydrogen bonds. The adjacent columns are connected through C_(methyl)_–H···O_(sulfo)_ hydrogen bonds between nimesulide anions to produce 3D framework (Fig. 9, Table S2).

### ADME analysis

Bioavailability Radar is one of results, which the Swiss ADME web tool is giving. It is prediction on six physicochemical properties, such as lipophilicity, size, polarity, insolubility, insaturation, flexibility^51^. Descriptors used for it, in comparison to pure nimesulide, are presented in Table 2.

**Table 2:** ADME analysis for nimesulide and compounds **1** and **2**.

Structure	ADME diagram	XLOGP3^a^	MW^b^	TPSA^c^	LOG S (ESOL)^d^	F. Csp^3 e^	NRB^f^
Nimesulide		2.60	308.31	109.60	− 3.48	0.08	5
**1**		2.88	381.45	97.57	− 4.03	0.29	5
**2**		4.35	437.55	97.57	− 5.00	0.43	9

^a^Parameter for lipophilicity calculations^52^.

^b^Molecular weight [g/mol].

^c^Topological polar surface area [Å^2^]^53^.

^d^Estimated solubility^54^.

^e^Ratio of sp^3^ hybridized carbons over the total amount of carbons in molecule.

^f^Number of rotatable bonds.

The pink area illustrates optimal range for six parameters. Nimesulide represents proper values for 5 properties. Lipophilicity, calculated by XLOGP3 is between − 7 and + 5 and size (MW) is between 150 and 500 g/mol. Other properties, such as polarity (TPSA) and solubility (logS), are in range 20–130 Å^2^ and not higher than 6, respectively. One of properties of nimesulide, saturation, which is described as a number of carbons in the sp^3^ hybridization—is out of range. The value of the parameter should be more than 0.25. All parameters for compounds **1** and **2** are in the range of the pink area, which means that substances are predicted to be orally bioavailable. Another parameter, referring to drug-likeness, is described by Lipinski’s rule of five^55^ from Pfizer and Bioavailability Score^56^ by Abbott. Both criteria, for all structures are in the acceptable range—Lipinski’s filter causing 0 violation and Bioavailability score is 0.55. Amount PAINS #alarm^57^ for all compounds is 0 alarm and the value of Synthetic accessibility^58^ for all structures is in an optimal range.

**Figure 10:**
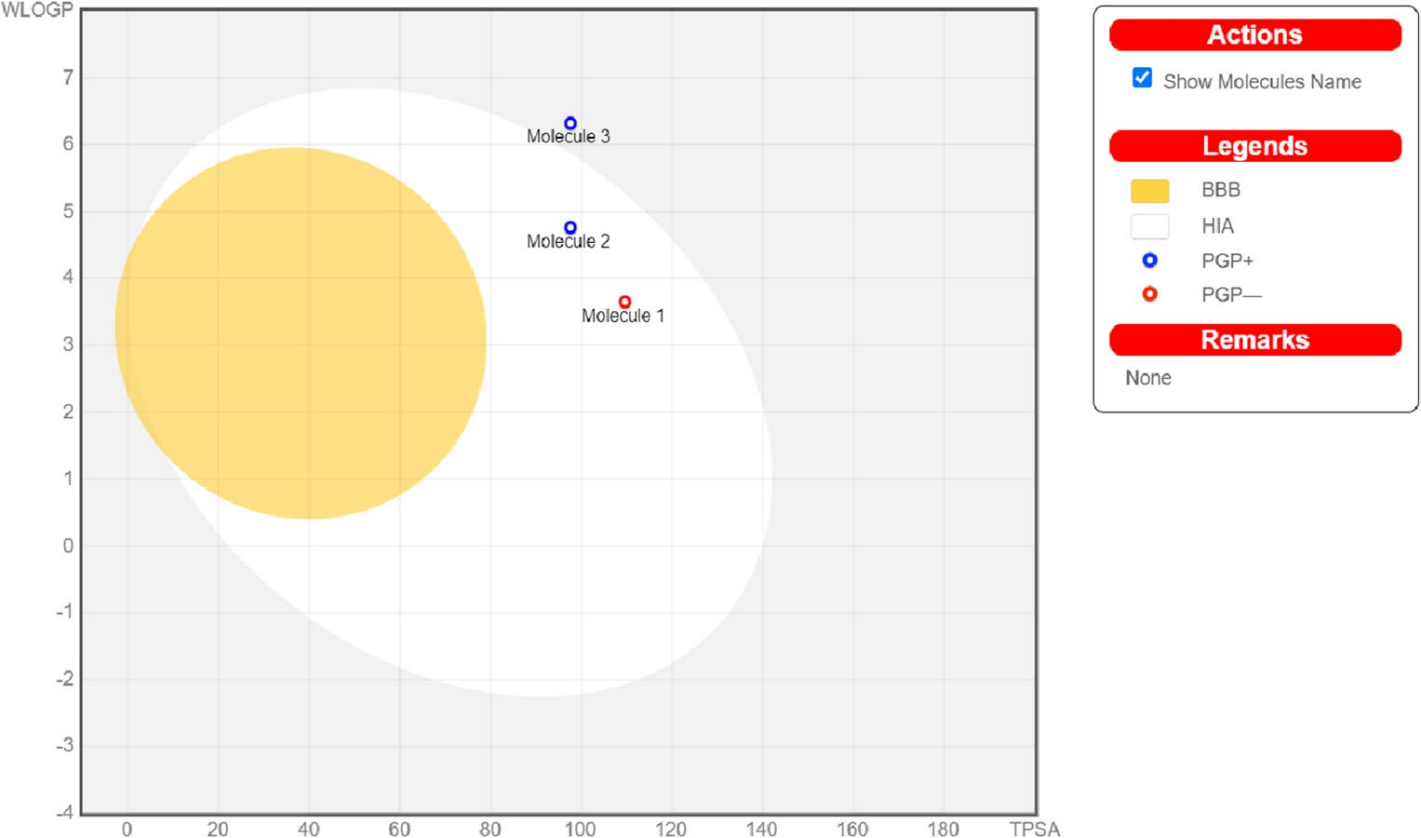
BOILED-Egg diagram for the nimesulide (Molecule 1) and compounds **1** (Molecule 2) and **2** (Molecule 3).

Method to predict at the same time two properties: the passive gastrointestinal absorption (HIA), brain access (BBB) is called BOILED-Egg. This model is based on two physicochemical parameters—WLOGP and TPSA. The BOILED-Egg plot (Fig. 10) contains 3 areas: the yellow one is assigned for most likely BBB permeation, the white one is a space for most likely HIA absorption molecules and the grey one stands for structures predicted to have weak absorption and low brain penetration. Yellow and white compartments are not mutually exclusive^59^. Occurred points are colored. Molecules, which are not effluated from the central nervous system by P-glycoprotein substrates (PGP-), are marked as red dots. Blue dots are for substances predicted to dispose from the central nervous system by P-glycoprotein substrates (PGP+)^60^. Nimesulide and compound **1** are predicted to be well-absorbed and not crossing the blood–brain barrier, which is confirmed in the literature for the nimesulide^61,62^, whereas compound **2** is predicted not to be absorbed and not accessing the brain. Compounds **1** and **2** are rid of the nervous system (PGP+), while nimesulide is not pumped-out by P-glycoprotein (PGP-). The Servis ProTox II has classified all molecules (nimesulide, compound **1** and **2**) into toxicity class 4 (predicted LD_50_: 997 mg/kg).

## Conclusion

The synthesis, spectral properties, thermal analysis, and structural characterization of tetramethylammonium (compound **1**) and tetraethylammonium (compound **2**) salts of nimesulide were described in this article. The MALDI-TOF, ^1^H NMR, ^13^C NMR and ATR-FTIR analyses confirm SCXRD study, that nimesulide exists in an ionized form in both studied compounds. MALDI-TOF mass spectra of the compounds **1** and **2** showed the peaks of nimesulide anion (found at *m/z* 307.045, and 307.008, respectively), and tetramethylammonium and tetraethylammonium cations (found at *m/z* 74.218 and *m/z* 130.198, respectively). A singlet at 10.2 ppm in the ^1^H NMR spectra of pure nimesulide corresponding to H–N proton of sulfonamide group, disappeared in the ^1^H NMR spectra of both title compounds due to the amide proton detachment. Likewise, formation of the salts resulted in a disappearance of N–H stretching band at 3278/cm in the ATR-FTIR spectra. The TG and DSC measurements confirmed that compound **1** melts at a temperature higher than nimesulide (179 °C), whereas the compound **2** melts at a temperature lower than nimesulide (113 °C). Compounds **1** and **2** crystallize in the monoclinic* P*2_1_/n space group, with one tetraalkylammonium cation and one nimesulide anion in the asymmetric unit, and their crystal structures are stabilized by C–H···O hydrogen bonds between ions. Additionally, structure of compound **1** is stabilized through π–π interactions, whereas in the crystal packing of compound **2** the C–H···π interactions between nimesulide anions playing an important role. Studies performed by SWISS ADME and ProTOX II tools, predict to be oral bioavailability of both salts obtained, and one of them (compound **1**) was predicted to be well-absorbed by digestive system. Both compounds were predicted to be classified into toxicity class 4 (predicted LD50: < 1000 mg/kg).

### Supplementary Information

Supplementary Tables.

## Data Availability

Crystallographic data for the structures reported in this article have been deposited at the Cambridge Crystallographic Data Centre, under deposition numbers No. CCDC 2281374 (1) and CCDC 2281375 (2). Copies of the data can be obtained free of charge via https://www.ccdc.cam.ac.uk/structures/. The authors declare that the data supporting the findings of this study are available within the paper and its Supplementary Information files. Should any raw data files be needed in another format they are available from the corresponding author upon reasonable request. Source data are provided with this paper.
