# Supplementary material for: The synthesis, thermal behaviour, spectral and structural characterization, and in silico prediction of pharmacokinetic parameters of tetraalkylammonium salts of non-steroidal anti-inflammatory drug nimesulide
Source: Sci Rep. 2023 Oct 12;13:17268. doi: 10.1038/s41598-023-44557-x (PMC10570311; doi:10.1038/s41598-023-44557-x)
Supplement: Supplementary file 1 — Supplementary Tables. [file 41598_2023_44557_MOESM1_ESM.docx]

**Electronic Supplementary Information**

**The synthesis, thermal behaviour, spectral and structural characterization, and *in silico* prediction of pharmacokinetic parameters of tetraalkylammonium salts of non-steroidal anti-inflammatory drug nimesulide**

Małgorzata Rybczyńska^*^ & Artur Sikorski^*^

University of Gdańsk, Faculty of Chemistry, W. Stwosza 63, 80-308 Gdańsk, Poland

*E-mail: malgorzata.rybczynska@phdstud.ug.edu.pl (M.R); artur.sikorski@ug.edu.pl (A.S.)

**Single-Crystal X-Ray Diffraction (SCXRD) measurements and structure refinement**

SCXRD data were collected on an Oxford Diffraction Gemini R ULTRA Ruby CCD diffractometer MoKα (*λ*_Mo_=0.71073 Å, T=293(2) K) (Table S1). All interactions were calculated using the PLATON program (Tables S2-S4).

**Table S1.** Crystal data and structure refinement for title compounds.

| **Compound** | | **1** | **2** |
| --- | --- | --- | --- |
| Chemical formula | | C_17_H_23_N_3_O_5_S | C_21_H_31_N_3_O_5_S |
| Formula weight/g·mol^-1^ | | 381.44 | 437.55 |
| Crystal system | | monoclinic | monoclinic |
| Space group | | *P*2_1_/n | *P*2_1_/n |
| *a*/Å | | 13.7425(18) | 12.0108(7) |
| *b*/Å | | 8.3439(9) | 11.5166(7) |
| *c*/Å | | 17.529(2) | 16.7784(9) |
| *α*/° | | 90 | 90 |
| *β*/° | | 108.752(14) | 97.564(5) |
| *γ*/° | | 90 | 90 |
| *V*/Å^3^ | | 1903.3(4) | 2300.6(2) |
| *Z* | | 4 | 4 |
| *T*/K | | 293(2) | 293(2) |
| *λ*_Mo_/Å | | 0.71073 | 0.71073 |
| *ρ_cal_*_c_/g·cm^–3^ | | 1.331 | 1.263 |
| *F(000)* | | 808 | 936 |
| Crystal size/mm^3^ | 0.41 × 0.20 × 0.09 | | 0.44 × 0.21 ×0.09 |
| µ/mm^-1^ | | 0.202 | 0.176 |
| *θ* range/° | | 3.30 - 25.00 | 3.63 - 25.00 |
| Completness *θ*/% | | 99.8 | 99.7 |
| Reflections collected | | 11775 | 15981 |
| Reflections unique | | 3328 [R_int_=0.0481] | 4043 [R_int_ = 0.0285] |
| Data/restraints/parameters | | 3328 / 66 / 269 | 4043 / 0 / 276 |
| Goodness of fit on *F^2^* | | 1.039 | 1.087 |
| Final R_1_ value (*I*>2σ(*I*)) | | 0.0785 | 0.0597 |
| Final *w*R_2_ value (*I*>2σ(*I*)) | | 0.1763 | 0.1605 |
| Final R_1_ value (all data) | | 0.1275 | 0.0767 |
| Final *w*R_2_ value (all data) | | 0.2084 | 0.1729 |
| Largest diff. peak/hole/e Å^−3^ | | 0.226/-0.227 | 0.499/-0.227 |
| CCDC number | | 2281374 | 2281375 |

**Table S2.** Hydrogen bonds geometry for compounds **1**-**2**.

| **Compound** | **D–H···A** | ***d*(D–H) [Å]** | ***d*(H···A) [Å]** | ***d*(D⋯A) (Å)** | **∠D–H⋯A (°)** |
| --- | --- | --- | --- | --- | --- |
| **1** | C25–H25B···O9  C24–H24A···O9^i^ | 0.96  0.96 | 2.58  2.46 | 3.540(7)  3.391(6) | 177  164 |
|  | C24–H24B···O21^ii^ | 0.96 | 2.60 | 3.490(8) | 155 |
|  | C23–H23A···O10^iii^ | 0.96 | 2.63 | 3.494(6) | 149 |
|  | C11–H11A···O9^iv^ | 0.96 | 2.64 | 3.514(6) | 152 |
| Symmetry code: (i) 3/2-x,1/2+y,1/2-z; (ii) 1-x,1-y,1-z; (iii) 3/2-x,-1/2+y,1/2-z; (iv) 1-x,1-y,-z. | | | | | |
| **2** | C25–H25A···O9 | 0.97 | 2.41 | 3.070(4) | 125 |
|  | C5–H5A···O21^i^ | 0.93 | 2.53 | 3.257(4) | 135 |
|  | C11–H11A···O20^ii^ | 0.96 | 2.64 | 3.514(6) | 152 |
|  | C25–H25B···O9^iii^ | 0.97 | 2.58 | 3.532(4) | 169 |
|  | C27–H27A···O10^ii^  C29–H29A···O10^iv^ | 0.97  0.97 | 2.57  2.53 | 3.400(4)  3.406(4) | 143  150 |
| Symmetry code: (ii) 1-x,1-y,1-z; (i) 3/2-x,1/2+y,1/2-z; (iii) 1-x,1-y,-z; (iv) 2-x, 1-y, 1-z. | | | | | |

**Table S3.** Geometry of π–π interactions for compound **1** (distance in Å and angle in degrees).

| Compound | CgI^a^ | CgJ^a^ | CgI···CgJ^b^ | Dihedral angle^c^ | Interplanar distance^d^ | Offset^e^ |
| --- | --- | --- | --- | --- | --- | --- |
| **1** | 2 | 2^v^ | 3.374(6) | 0.0(5) | 3.025(4) | 1.496 |
| Symmetry code: (v) 1-x,-y,1-z. | | | | | | |

^a^Cg 2– centre of gravity of the rings delineated by the atoms: C13/C14/C15/C16/C17/C18.

^b^Cg···Cg – distance between ring centroids.

^c^Dihedral angle – angle between the mean planes of CgI and CgJ.

^d^Interplanar distance – perpendicular distance from CgI to ring J.

^e^Offset – perpendicular distance from ring I to ring J.

**Table S4**. Geometry of C–H···π interaction for compound **2**.

| Compound | X–H···Cg(J) | *d*(H···Cg)[Å] | *d*(X···Cg) [Å] | ∠X–H⋯Cg (°) | |
| --- | --- | --- | --- | --- | --- |
| **2** | C17–H17A···Cg1^v^ | 2.95 | 3.762 | 146 |  |
| Symmetry code: (v) 1-x,-y,1-z. | | | | | |

H···Cg – distance between hydrogen to ring centroid.

Cg1 – centre of gravity of the rings delineated by the atoms: C1/C2/C3/C4/C5/C6.
